# Effects of mixed hardwoods dust on respiratory function and blood immunoglobulin levels in wood workers

**DOI:** 10.1016/j.heliyon.2024.e26358

**Published:** 2024-02-15

**Authors:** Isaac E. Ennin, Festus K. Adzaku, Daniel Dodoo, Raymond Saa-Eru Maalman

**Affiliations:** a Department of Physician Assistant Studies, School of Medicine and Health Sciences, Central University, Ghana; b Department of Physiology, University of Ghana Medical School, College of Health Sciences, University of Ghana, Ghana; c Department of Immunology, Noguchi Memorial Institute of Medical Research, College of Health Sciences, University of Ghana, Ghana; d Department of Basic Medical Sciences, School of Medicine University of Health and Allied Sciences, Ghana

**Keywords:** Mixed tropical hard, IgE, IgG, Lung volumes and airflow rate, Respiratory symptoms

## Abstract

**Background:**

Occupational exposure to wood dust, generated by various individual wood species, both softwood and hardwood, has been extensively documented as a causative factor for reduced lung function, frequent respiratory symptoms, and increased immunological responses in wood workers. This study explores the impact of wood dust from mixed tropical hardwood species on lung function, respiratory symptoms, and Immunoglobulin (Ig) E and G levels.

**Methods:**

A cross-sectional study was conducted among wood workers at the Accra Timber Market and a control group from the University of Ghana. Particulate matter (PM) was sampled using a Minivol Sampler set to a flow rate of 5 l/min. Respiratory symptoms were assessed using questions adapted from the British Medical Research Council (MRC) questionnaire (1960). Lung volumes and airflow rates were measured using a spirometer. Total serum IgE and IgG levels were quantified using ELISA.

**Results:**

No significant differences were observed between the wood workers and the controls for demographic variables. Wood workers exhibited a significantly higher prevalence of respiratory symptoms, particularly rhinitis, with many reporting the absence of symptoms during holidays. Lung function parameters (VC, FEV1, FEV1%, PEFR, and FEF25-75%) were significantly reduced (p < 0.05) in wood workers. A significant negative correlation was noted between lung function parameters and years of exposure to wood dust. Wood workers showed significantly elevated levels (p < 0.05) of IgG and IgE.

**Conclusion:**

The study findings suggest that exposure to mixed tropical hardwood dust induces elevated blood IgE and IgG levels, along with non-allergic respiratory function abnormalities.

## 1 Background

Exposure to wood dust has been linked to various external and internal health issues [1]. Reported adverse health effects associated with wood dust exposure encompass upper respiratory problems, eye irritation, dermatitis, dry cough, malaise, chronic bronchitis, shortness of breath, chest pain, conjunctivitis, rhinitis, occupational asthma, allergic alveolitis, headache, cancer, and lung function deficits [2,3] Wood workers exposed to wood dust have also demonstrated a high prevalence of regular respiratory symptoms, including cough, sneezing, dyspnea, chest pain, and persistent wheezing [1]. The concentration of wood dust in woodworking shops is suggested to contribute to negative respiratory health effects.

The Occupational Exposure Limit (OEL), representing the average dust exposure measured over an 8-h work period, varies with different types of wood due to varying levels of toxicity. OEL values range from 0.5 to 5.0 mg/m3 (total dust) in countries such as Canada, New Zealand, Australia, and the European Union, while it is 15 mg/m3 in Nigeria [1,4].

Respiratory dysfunction associated with exposure to wood dust, generated from both softwood and hardwood, has been extensively documented in wood workers, manifesting as reduced lung function, including forced vital capacity (FVC), forced expiratory volume in 1 s (FEV1), forced expiratory volume ratio (FEV1/FVC), maximum voluntary ventilation (MVV), and peak expiratory flow rate (PEFR), as well as FEF_25–75%_ and increased prevalence of respiratory symptoms [[5], [6], [7], [8], [9]]. Increased prevalence of respiratory symptoms, such as frequent wheezing, sneezing, rhinitis, loss of voice, shortness of breath at rest, shortness of breath during exercise, difficulty in breathing, and chest pain, has also been reported in some wood workers in prior studies [7,[9], [10], [11], [12]]. Immunological responses observed in wood workers exposed to wood dust from either single softwood species or hardwood species have shown higher concentrations of immunoglobulin G and E relative to matched controls, suggesting involvement of allergic reaction pathways in the pathophysiology of wood dust-related respiratory health effects [9,[13], [14], [15]].

In the Accra Timber Market of Ghana, various tropical hardwood species, such as Khaya anthotheca (African mahogany), Milicia excelsa (odum), Albizia ferruginea (awiemfosamina), Antiaris toxicaria (kyen-kyen), Ceiba pentandra (onyina), and Terminalia ivorensis (emire), are processed, generating mixed-species wood dust. Health effects of wood dust have been reported to vary with tree species, and this variability is attributed to different chemical compounds naturally occurring in the wood [2,12]. Extractives from hardwood exhibit varying effects on humans, ranging from pharmacologically beneficial to physiologically deleterious, including anti-inflammatory and anti-mutagenic properties, as well as proinflammatory and cancer-promoting characteristics [16,17]. The anti-inflammatory effects of extractive compounds such as flavonoids, tannins, coumarins, quinones, and others from tropical hardwood have been documented [18]. Additionally, occupational asthma due to exposure to specific hardwood species, such as Western red cedar, iroko, obeche, ash wood, teak, cedar of Lebanon, oak, mahogany, and redwood, is well established [19,20].

The majority of Ghanaian wood workers operate without any protective equipment, exposing them to occupational hazards associated with woodwork, posing a major concern for public health. Despite this, limited research has focused on the impact of wood dust from a combination of different wood species on the respiratory health of Ghanaian wood workers. This study aimed to determine the association between respiratory systems, lung function and duration of exposure to the blood immunoglobulin levels in wood workers exposed to mixed tropical hardwood dust in Ghana.

## 2 Method

### 2.1 Study site, design and study population

A cross-sectional study involving wood workers from the Accra Timber Market and a control group from the University of Ghana, College of Health Sciences, Korle-Bu, was conducted to assess the correlation between lung function, respiratory symptoms, and IgG/IgE concentrations. The study took place from October 2008 to March 2009. Accra, situated in the southeastern part of Ghana and bordered by the Gulf of Guinea to the south, experiences two rainy seasons annually, with an average rainfall of 790 mm, an average temperature ranging from 24.6 °C to 31.1 °C, and an average humidity of 81%. Serving as the capital, Accra is a metropolitan city with numerous privately-owned small-scale wood factories, particularly concentrated in the Accra Timber Market.

Most shops in the timber market are characterized by temporary shelters supported by 6–8 wooden poles and roofs made of corrugated iron sheets. These shops lack walls, while some are partially enclosed, and a few have wooden board walls separating one shop from another. Some may have cement block walls enclosing the entire shop. Each shop may house 2–4 different types of machines, with 6–15 workers and a small office space for supervisors or cashiers. Ventilation in the timber market is generally poor, with some shops having short wooden poles supporting the roof, others partially enclosed, and wood shavings and dust scattered throughout the market.

Cluster and convenient sampling were used to sample one hundred and fifty (150) male adult sawmill workers who were exposed to mixed tropical hardwood dust 12 h a day (6:00am - 6:00pm) at the Accra Timber Market. Participants were interviewed and recruited based on eligibility criteria. Comprehensive histories were taken to determine inclusion in the study. Similarly, 120 adult workers with no history of exposure to occupational air pollutants were recruited from the University of Ghana, College of Health Sciences, Korle-Bu, to serve as the control group.

## 3 Selection criteria

### 3.1 Inclusion criteria

A male worker of age between 20 and 60 years, who had worked for at least 6 months and has not been exposed to any occupational pollutant other than wood dust (for wood workers) without clinical abnormalities of the vertebral column, the thoracic cage and with no history of smoking (based on self-reporting medical history).

### 3.2 Exclusion criteria

Workers not willing to participate in the study. Workers previously exposed to any occupational pollutants and workers with history of smoking (based on self-reporting medical history).

## 4 Data collection

### 4.1 Interviews

Respiratory symptoms questions adapted from the British Medical Research Council (MRC) questionnaire (1960) were used. Components of the questionnaires included variables such as frequent respiratory symptoms medical history, occupational history, smoking history, alcohol intake, exposure to other industrial pollutants, (apart from wood dust) years of exposure to wood dust and status of respiratory symptoms away from workplace were answered by the participant (based on self-reporting medical history). Three National Service personnel with first degree in Human Biology together with the principal researcher (all trained to collect data) were engaged in the interviews. Each subject was assisted in answering the questions by interview in English or Twi language.

### 4.2 Lung volumes and airflow measurement

The lung volumes and airflow of the participants were measured using a spirometer (Alpha Vitalograph, Buckingham England) which had been standardized according to the specifications of the American Thoracic Society. Measurements were done with participant in a standing position. There were two test manoeuvres conducted in each lung volume and airflow test: the static and dynamic.

Prior to each measurements participants were instructed to abstain from alcohol, performing exercise and eating large meal within 2 h before the test. Each participant was asked to loosen any tight-fitting clothing or belt just before the test was performed [21]. The test was demonstrated to each of the participants before asked to perform.

The static test begun by instructing the participant on the manoeuvre, this was followed with a demonstration of the technique. The participants were made to understand that they must completely fill and empty their lungs. The test was performed with a participant using a mouthpiece and wearing nose clip. He was then instructed to inhale maximally and hold his breathe while he inserted the mouthpiece just past the front teeth, with the mouthpiece lying on the tongue and the lips forming a sealed around it. He exhales the air slowly and evenly until there was no volume change for 1 s period (not unduly slow). This manoeuvre measured the vital capacity (VC).

### 4.3 Dynamic manoeuvre

There are three distinct phases to this manoeuvre as follows (1) maximal inspiration (2) a blast of exhalation and (3) continued complete exhalation to the end of test. As in the static manoeuvre the dynamic manoeuvre begun by instructing the subject on the manoeuvre and followed with a demonstration of the technique. The subject was made to understand that he must completely fill and empty his lungs. The test was performed with the subject using a mouthpiece and wearing nose clip. The participant was instructed to inhale fully and rapidly with a pause of less than 1s at total lung capacity while he inserted the mouthpiece and exhale maximally and forcefully until no more air could be expelled while maintaining an upright posture to the end. This manoeuvre measured the Forced Vital Capacity (FVC), Forced Expiratory Volume in 1 s (FEV_1_), Forced Expiratory Volume Ration (FEVR), Peak Expiratory Flow Rate (PEFR), and Forced Expiratory Fraction 25–75% (FEF_25–75%_) of the subjects.

### 4.4 Quality control for spirometry

Each participant used a new disposable mouthpiece, and each manoeuvre was repeated three times while the best performance was used for the data analysis, each after adequate rest for the participant. Specific measurements from the participant's test were compared to standard predicted values stored in the spirometer. All the lung volumes were measured at a fixed time of the day (9:00–13:00 h) to minimize diurnal variation [22]. The precise technique in executing various lung function tests for the study was based on the operational manual of the instrument. The instrument was calibrated daily and operated within the ambient temperature range of 24–28 °C.

### 4.5 Collection of blood samples

Each participant donated, 5 ml of blood, which was collected by venipuncture of the median cubital veins. The blood samples were then put into vacutainer tubes and placed in ice in an insulated box. The blood samples were later spun in a refrigerated bench top centrifuge (DENLEY, BR401) at 1000×*g* centrifugal force for 5 min and at 4 °C. The sera formed was pipetted into 1000 μl Eppendorf tubes in ice and stored at - 80 °C until time of assay.

### 4.6 Measurement of serum IgE and IgG concentrations

The immunoglobulin E (IgE) and G (IgG) levels were measured at the Department of Immunology, NMIMR, College of Health Sciences, University of Ghana, using the Enzyme-Linked ImmunoSorbent Assay (ELISA). Total serum IgE and IgG were measured in flat-bottomed 96-well micro-ELISA plate (Nunc Denmark).

The ELISA plates were initially coated at 50 μl/well with affinity purified goat anti-human IgE (Vector Laboratory, Burlington, Ca USA) at a concentration of 5 μg/l in coating buffer (pH 9.6) and incubated overnight at 4 °C.

The plates were then washed 4 times with washing buffer (pH 7.4) and blocked with 3% skimmed milk in washing buffer at 200 μl/well and incubated at 37 °C for 3 h.

The plates were again washed 4 times with washing buffer. The test sera diluted at 1:5000 in a diluent were added at 50 μl/well and Biotinylated goat anti-human IgE (Vector Laboratory, Burlington, Ca USA) diluted at 1:1000 in a diluent were added at 50 μl/well and incubated overnight at 4 °C.

The ELISA plates were then washed 6 times with washing buffer, followed by the addition of an enzyme label Avidin peroxidase conjugate (Sigma Chemical Co.) diluted at 1:1000 in a diluent at 50 μl/well and incubated at room temperature for 1 h. The plates were washed 8 times after the incubation.

Finally, the substrate TMB (KEM EN TEC Diagnostic) was added at 100 μl/well and incubated in the dark for 25 min. Thereafter 50 μl/well of 0.2 M dilute H_2_SO_4_ was added to terminate the reaction.

The optical density values were read in a Multiskan Ex (Thermo Electron Corporation Finland) microplate reader at 450 nm. Total IgE concentrations were calculated from standard curves obtained by including the coated plates with eight dilutions (3125, 781.3, 195.3, 48.8, 12.2, 3.1, 0.8 and 0.2) pg/ml of human serum IgE (Immunology Consultant Laboratory) in the diluent.

### 4.7 Measurement of serum IgG concentration

The ELISA plates were coated at 50 μl/well with affinity purified goat anti-human IgG (Vector Laboratory, Burlington, Ca., USA) at a concentration of 10 μg/l in coating buffer (pH 9.6) and incubated overnight at 4 °C.

The ELISA plates were then washed 2 times with washing buffer (pH 7.4). The plates were then blocked with 3% skimmed milk in washing buffer at 200 μl/well and incubated at room temperature for 1 h on a shaker. The plates were again washed 2 times with washing buffer, the test sera diluted at 1:9000 in a diluent were added at 50 μl/well and incubated at 4 °C overnight.

Horseradish peroxidase (HRP) diluted at 1:3000 in a diluent were added at 50 μl/well and incubated for 1 h at room temperature on a shaker. The plates were then washed 4 times with washing buffer.

Finally, the substrate TMB was added at 100 μl/well and incubated in the dark for 25 min. Thereafter 50 μl/well of 0.5 M dilute H_2_SO_4_ was then added to terminate the reaction.

The optical density values were read in a Multiskan Ex (Thermo Electron Corporation Finland) microplate reader at 450 nm. Total IgG concentrations were calculated from standard curves obtained by including the coated plates with eight dilutions (100.0, 50.0, 25.0, 12.5, 6.3, 3.1, 1.6, and 0.8) ng/ml of human serum IgG in the diluent.

### 4.8 Quality control for ELISA

The best practices were followed to obtain accurate and consistent data, and data analysis: The samples and standards were run in duplicate. A control sample with a known concentration and a blank sample (buffer and water) were run on each plate, similarly, a standard curve was plotted on each plate. The samples were diluted to fall within the linear range of the standard curve. A 4-parameter algorithm was used for the best standard curve fit. Also, the background absorbance was subtracted using the blank and the dilution factor was multiplied by the absorbance to obtain the final concentration. The standard deviation (SD), mean (X) and coefficient of variation (CV) (CV between the original and duplicate was 16.13%) were calculated.

## 5 Data analysis

At the end of the study, SPSS statistical package version 20 was used to analyze the data. The level of statistical significance was established at a p-value <0.05. Statistical analysis was conducted using an unpaired *t*-test to compare the demographic indices, lung volumes and airflow values (VC, FVC, FEV1, FEV1%, PEFR, FEF_25–75%_) and sera immunoglobulins (IgE and IgG) between wood workers and controls. Using the Pearson's correlation coefficient: Each of the pulmonary function indices was correlated with the years of work (duration of exposure) and the immunoglobulins. Similarly, the respiratory symptoms were correlated with the duration of exposure and the immunoglobulins. Also, the immunoglobulins were correlated with duration of exposure of the wood workers.

### 5.1 Ethical issue

All participants gave informed consent before taking part in the survey and the study was approved by the University of Ghana Medical School Protocol and Ethics Committee of the University of Ghana with reference number MS-ET/M3.3-P5/2007–08.

## 6 Result

One hundred and fifty non-smoking wood workers were selected from the Accra Timber Market for the study. Similarly of the 120 unexposed workers selected from the College of Health Sciences, University of Ghana Medical School, Korle-Bu for the controls group. Altogether, 104 male wood workers and 104 unexposed male workers (controls) finally participated in the study. The results obtained from the data analysis are presented as follows (the demographic data and lung function tests results have been published in an earlier publication [19].

### 6.1 Demographic data of wood workers and controls compared

Table 1 shows that, there were no significant differences between the means of the wood workers and the controls for all the demographic variables: Age (p = 0.713), Height (p = 0.880), Weight (p = 0.182), and Years of work (p = 0.797).

**Table 1:** Demographic data of wood workers and controls compared.

Variables	Wood workers mean ± SEM n = 104	Controls Mean ± SEM n = 104	*p* value
Age (yr.)	37.36 ± 1.03	37.92 ± 1.14	0.713
Height (cm)	169.47 ± 0.70	169.33 ± 0.64	0.880
Weight (kg)	72.25 ± 0.69	70.26 ± 1.32	0.182
Years of work	12.41 ± 0.915	12.04 ± 1.141	0.797

Significance at p-value <0.05. were based on unpaired *t*-test [19].

Prevalence of respiratory symptoms in wood workers and controls at work place.

Table 2 presents the prevalence of respiratory symptoms was significantly different between the wood workers and the controls. The wood workers showed a significantly higher prevalence (p < 0.01) of all the respiratory symptoms. Significantly high proportions of the wood workers reported of frequent sneeze, frequent cough, breathlessness at rest, frequent loss of voice, breathlessness after work, frequent rhinitis and chest pain since they started working in the wood industry.

**Table 2:** Prevalence of respiratory symptoms in wood workers and controls at work place.

Respiratory symptoms	Wood workers (%) n = 104	Controls (%) n = 100	p-value
Wheeze	35	4	<0.01*
Sneeze	58	8	<0.01*
Cough	46	4	<0.01*
Breathlessness at rest	30	1	<0.01*
Loss of voice	29	2	<0.01*
Breathlessness after work	21	2	<0.01*
Rhinitis	61	1	<0.01*
Chest pain	21	2	<0.01*

P-values; *p < 0.05 were based on Fischer test.

### 6.2 Status of respiratory symptoms away from workplace

Most of the wood workers reported of absence of respiratory symptom when they are away from the workplace during holidays as shown in Table 3.

**Table 3:** Status of respiratory symptoms away from work place.

Respiratory symptoms	Freq.	%
Present	93	89
Absent	11	11

### 6.3 Comparison of lung volumes and airflow values of the wood workers and controls

Comparing the lung volumes and airflow values between the wood workers and controls. There were significant reductions (p < 0.05) in the means of the lung volumes and the airflow values (VC, FEV_1_, FEV_1_%, PEFR and FEF_25–75%_) of the wood workers relative to the controls. There was no significant difference in the means of the FVC for the wood workers and the controls as shown in Table 4.

**Table 4:** Comparison of lung volume and airflow values of wood workers and controls.

Parameter	Wood workers mean ± SEM n = 104	Controls Mean ± SEM n = 104	*p* value
VC	3.32 ± 0.06 (82.93)	3.55 ± 0.06 (88.58)	0.008*
FVC	3.46 ± 0.08 (89.77)	3.63 ± 0.07 (94.77)	0.095
FEV_1_	2.58 ± 0.07 (79.29)	2.90 ± 0.06 (91.00)	0.001*
FEV_1_%	73.12 ± 2.03 (90.39)	79.13 ± 1.01 (98.07)	0.001*
PEFR(Ls^−^)	305.43 ± 13.13 (64.40)	392.3 ± 11.3 (83.88)	0.000*
FEF _25–75%_ (Ls^−^)	2.52 ± 0.11 (65.58)	3.00 ± 0.10 (78.96)	0.002*

P-values; * p-value <0.05 were based on unpaired *t*-test [19].

### 6.4 Comparison of blood sera total IgG and IgE concentrations in wood workers and controls

Comparing the mean concentrations of the blood sera total IgG and IgE of wood workers and controls. The mean concentrations of IgG and IgE were different in the wood workers (8.86 mg/ml, 0.69 μg/ml) and the controls (5.36 mg/ml, 0.28 μg/ml) respectively. The wood workers had significantly higher mean concentrations of IgG (p = 0.0002) and IgE (p = 0.0011).

### 6.5 Correlation of respiratory symptoms with IgE, IgG and years of exposure of wood workers

Table 5 shows the respiratory symptoms correlation with IgE, IgG and years of exposure.

**Table 5:** Correlation of respiratory symptoms with IgE, IgG and years of exposure of wood workers.

	IgE	IgG	Years of exposure
**Wheeze**	0.0881	0.1611	0.0738
	0.4035	0.1251	0.4845 (NS)
**Sneeze**	0.0953	0.1599	0.1233
	0.3663	0.1278	0.2415 (NS)
**Cough**	−0.0668	0.1343	−0.0902
	0.5272	0.2018	0.3925 (NS)
**Rhinitis**	**−0.2485**	0.129	0.1633
	0.0169*	0.2204	0.1198 (NS)

P-values; *p < 0.05 were based on Pearson correlation coefficient, NS- not significant.

Except for rhinitis which showed significant negative correlation with IgE, the other respiratory symptoms showed insignificant association with both IgE and IgG.

The respiratory symptoms showed no significant correlation with the years of exposure of the wood workers.

### 6.6 Correlation of lung volumes and airflow values with IgE, IgG and years of exposure in wood workers and control group

There was no significant correlation between the lung function values with the IgE levels for both the wood workers and the control. However, the lung volume VC had a significant positive correlation with the IgG in the wood workers but no significant correlation in the controls. Implying increase in VC is associate with increasing IgG in the wood workers.

The lung function values; FVC, FEV_1_, PEFR and FEF_25-75_ showed significant negative correlation with the years of exposure in both the wood workers and the controls. Implying, increase years of exposure is associated with reduction in lung function values. The lung volume VC showed significant negative correlation with the years of work in the control but this was not significant in wood workers as shown in Table 6.

**Table 6:** Correlation of lung volumes and airflow rate with IgE, IgG and years of work for wood workers and controls.

	Wood workers			Control		
	IgE	IgG	Years of work	IgE	IgG	Years of work
**VC**	0.1212	**0.3556**	−0.1654	0.0307	−0.1888	**−0.4342**
	0.2499	0.0005*	0.115	0.7962	0.1098	0.0001*
**FVC**	0.1196	0.1883	**−0.2438**	0.0441	−0.1587	**−0.3744**
	0.2563	0.0723	0.0192*	0.7109	0.1798	0.0011*
**FEV**_**1**_	0.0208	0.1702	**−0.3272**	0.2224	−0.1166	**−0.4478**
	0.8442	0.1047	0.0015*	0.8519	0.3257	0.0001*
**FER**	−0.0617	0.0015	**0.2244**	−0.0014	0.15	−0.1759
	0.5589	0.9885	0.0315*	0.9908	0.2052	0.1366
**PEFR**	0.0001	0.0054	**−0.2806**	−0.0375	−0.1039	−0.1954
	0.9994	0.9593	0.0067*	0.7527	0.3815	0.0976
**FEF**	−0.0396	0.0778	**−0.2793**	−0.0839	0.02001	**−0.2637**
	0.7077	0.4609	0.007*	0.4801	0.8665	0.0242*

P-values; *p < 0.05 were based on Pearson correlation coefficient, significant correlation values in bold.

### 6.7 Correlation of IgE and IgG levels with years of exposure of the wood workers

Table 7 shows IgE and IgG correlation with years of exposure of wood workers. There was no significant association between the immunoglobulins and the years of exposure of the wood workers.

**Table 7:** Correlation of IgE, and IgG with years of exposure of wood workers.

Immunoglobulins	Years of exposure	
**IgE**	−0.0169	
	0.873 (NS)	
**IgG**	−0.0723	
	0.4932 (NS)	

P-values; p < 0.05 were based on the Pearson correlation coefficient, NS-not significant.

## 7 Discussion

The health effects of wood dust have been reported to vary with tree species, and the observed variability in health effects between species has been attributed to different chemical compounds naturally occurring in the wood [13,16]. Extractives from wood have been found to elicit varying effects on humans, ranging from pharmacologically beneficial to physiologically deleterious. These effects span from anti-inflammatory and anti-mutagenic properties to proinflammatory and cancer-promoting characteristics [17,18]. The anti-inflammatory effects of extractive compounds, such as flavonoids, tannins, coumarins, quinones, and others, have been well-documented [[23], [24], [25]]. Occupational asthma resulting from exposure to specific tree species, such as Western red cedar, iroko, obeche, ash wood, teak, cedar of Lebanon, oak, mahogany, and redwood, is well-established [[26], [27]].

In this current study, wood workers were subjected to wood dust emanating from a variety of wood species simultaneously, and notably, without employing any personal protective equipment. The demographic variables showed no significant differences between the exposed wood workers and the control group. The elevated concentration of wood dust in both the workshop and the market can be ascribed to inadequate ventilation in the timber market. Some shops featured roofs supported by short wooden poles, while others were only partially enclosed. Additionally, heaps of wood shavings and dust were scattered throughout the market, with no provision of dust extraction devices in the workshops.

The wood workers in this study exhibited a significantly higher prevalence of respiratory symptoms compared to the control group, aligning with findings from other studies [8,9,11,20]. The most frequently reported respiratory symptom in this study was rhinitis, accounting for 61% of cases, a trend consistent with the observations made by Jacobsen et al. [20]. Tobin et al. [25] also documented a notable prevalence of respiratory symptoms, particularly cough and phlegm, among tropical hardwood workers. However, Enarson and Chan-Yeung [28] highlighted in their studies that exposure to both softwood and hardwood dust is associated with various non-malignant health effects, including allergic rhinitis, chronic bronchitis, and allergic asthma.

Bronchial hyper-reactivity was observed in woodworkers exposed to oak and beech dust levels between 2.96 and 12.74 mg/m^3^ [29]. In addition, Tobin et al. [25] observed prevalence of respiratory symptoms particularly cough and phlegm among tropical hardwood workers with exposure level of 1.39 mg/m^3^. However, the exposure level of the present study compared with previous studies is lower.

Contrary to previous studies [20,30,31], this study did not find any significant association between respiratory symptoms and the duration of exposure for the wood workers. This suggests that respiratory symptoms only manifest when wood workers are exposed to wood dust in the workplace. This observation is further supported by the majority of wood workers reporting an absence of respiratory symptoms when away from the workplace.

The heightened prevalence of respiratory symptoms among wood workers may be attributed to exposure to elevated ambient air wood dust concentrations without the use of personal protective equipment. This finding can be attributed to the presence of irritant molecules or mechanical irritation caused by wood dust exposure in wood workers. Reduction in lung volume and airflow rates has been associated with lung function abnormalities, leading to inflammation and narrowing of the airways. Spirometry, a physiological test measuring lung volume and airflow rate, included parameters such as VC, FVC, FEV1, FER, PEFR, and FEF25-75% in this study. Except for FVC, all other spirometry parameters measured showed a significant reduction in wood workers compared to the control group. This aligns with observations by Uzoma et al. [30], Mogal et al. [8], and Patil and Krishnan [7], suggesting that wood workers exposed to mixed tropical hardwood dust may experience lung function abnormalities due to high wood dust exposure without personal protective equipment.

Furthermore, with the exception of VC, all lung function parameters measured exhibited a significantly negative correlation with the years of exposure for wood workers to wood dust. This indicates a decrease in lung function parameters (FVC, FEV1, FEV1/FVC, PEFR, FEF) with increasing years of exposure to wood dust, consistent with findings in other studies [8,32]. This suggests that prolonged exposure to mixed tropical hardwood dust may induce inflammation, leading to airway narrowing and the subsequent reduction in lung function parameters.

Interestingly, this study also noted a reduction in lung volumes and airflow values with increasing years of work in the control group. This implies that the decrease in lung function is not solely attributed to wood dust exposure but may be influenced by the cumulative years of work. Furthermore, as it is well documented, lung function reduces with increase in age [32,22]. This then suggests that age increase in the woodworkers rather than wood dust may have greater impact on the reduction in lung function. This is supported by observations from Borm et al. [33], reporting no reduction in lung function, respiratory symptoms, or inflammation of the upper airway tract in wood workers exposed to meranti wood dust. Similarly, Bohadana et al. [34] found a 0% prevalence of chronic bronchitis, and Glindmeyer et al. [22], in a 5-year follow-up study, reported no cases of COPD in the wood workers they investigated. Baran et al. [35] noted no obstructive or restrictive lung defects, and another study by Bohadana et al. [34] found no obstructive defects in the wood workers they studied.

Several studies have demonstrated IgE responses against various types of wood, including pine [13,[36], [37]]. Mechanical irritation of the airways by dust particles with high molecular weight (greater than or equal to 5000 Da) has been found to stimulate the production of specific IgE [13]. Besides IgE-mediated sensitization, several other mechanisms are possible in the development of bronchial asthma in wood workers [18,19]. In their study, Ricciardi et al. [18] observed that iroko wood may induce occupational asthma through immunological mechanisms other than IgE-mediated immediate hypersensitive reactions.

In the present study, the total serum IgE level was significantly higher in wood workers than in the controls, consistent with the findings of Mogal et al. [9]. However, this contrasts with the findings of Uzoma et al. [30], who found no significant difference in IgE levels between grain mill workers exposed to dust and the controls. The rise in IgE levels in wood workers in the present study was found to have no significant association with lung function parameters and all respiratory symptoms, except for rhinitis, which showed a significant negative association. This suggests that the reduced lung function values and frequent respiratory symptoms in wood workers may be induced by a non-allergic mechanism.

Immunoglobulin G has been shown to play a role in Th2-mediated allergic inflammation and is associated with allergic reactions in the airway induced by foreign antigens, such as bacterial and fungal spores [31,37]. In the present study, total serum IgG levels were higher in wood workers than in the controls, consistent with the observations of Uzoma et al. [30] in grain mill workers who also had a decrease in FVC and FEV1 % predicted values. However, the increase in IgG levels in wood workers in the present study did not show any significant association with lung volume, airflow rates, or respiratory symptoms, suggesting that no allergic reaction is involved in abnormalities in lung function.

## 8 Limitation

Personal sampling of wood dust for the wood workers could not be performed for lack of sampling device.

The participants in this study as in most cross-sectional studies might represent a survival population, so subject with more disabling symptoms might have changed jobs. This could have resulted in inconsistency observed in respiratory symptoms association with lung function defects and years of exposure.

There were not enough financial resources to purchase some consumables items such as mouthpieces, thermal paper, bronchodilators and reagent kits in order to do further relevant investigations as well as enroll more participants.

## 9 Conclusion

Exposure to mixed tropical hardwood dust may induce frequent respiratory symptoms and diminished lung function values in wood workers through non-allergic mechanisms. Mechanical irritation by the wood dust may contribute to the onset of respiratory symptoms. Additionally, the cumulative increase in years of exposure to wood dust, as opposed to wood dust alone, appears to be linked to the decline in lung function. Also, increase in age of wood workers contribute immensely to the reduction of lung function.

## Data availability statement

The datasets used and/or analysed during the current study are not publicly available but are available from the first author and corresponding author on reasonable request.

## CRediT authorship contribution statement

**Isaac E. Ennin:** Writing – original draft, Methodology, Data curation, Conceptualization. **Festus K. Adzaku:** Writing – review & editing, Supervision, Conceptualization. **Daniel Dodoo:** Writing – review & editing, Validation, Supervision, Methodology. **Raymond Saa-Eru Maalman:** Writing – review & editing, Writing – original draft, Formal analysis.

## Declaration of competing interest

The authors declare that they have no known competing financial interests or personal relationships that could have appeared to influence the work reported in this paper.
