# Supplementary material for: Effects of mixed hardwoods dust on respiratory function and blood immunoglobulin levels in wood workers
Source: Heliyon. 2024 Feb 15;10(4):e26358. doi: 10.1016/j.heliyon.2024.e26358 (PMC10884841; doi:10.1016/j.heliyon.2024.e26358)
Supplement: Multimedia component 1 [file mmc1.docx]

**SUBJECTS INFORMED CONSENT FORM and QUESTIONNAIRE**

PRINCIPAL INVESTIGATOR: Isaac Ekow Ennin

SUPERVISORS: Prof. Festus.K. Adzaku And Dr. Daniel Dodoo

ORGANIZATION: Department Of Physiology Ugms-Chs

SPONSOR: College Of Health Sciences

PROJECT TITLE: The Effect Of Wood Dust On Lung Function Of Wood Workers In The Accra Timber Market

**Introduction**

The nature of your work causes the emission of wood dust (containing toxics, chemical irritant, physical irritant and allergic sensitizers) into the atmosphere. Studies have shown that some people experience dry cough, shortness of breath, chest pain chronic bronchitis, asthma, headache, rhinitis, dermatitis and conjunctivitis among others due to the emissions.

The aim of this study is to find out whether the wood dust you breathe in everyday as you work has any damaging effect on your lungs using the spirometry.

Spirometry is the most basic and frequently performed test to measure lung function the results are used to assess the health status of your lungs. Repeated spirometric testing often is used for surveillance of workers at risk of developing occupational lung disease.

**Voluntariness**

You are being asked to take part in this study by volunteering to be a subject. You are to understand that:

1. Taking part in this research is entirely voluntary.
2. We will take the following measurements in the Physiology department of the Medical School: lung volumes, height and weight. One tablespoonful of blood will also be drawn from your body to measure the level of some substances. This amount of blood is not very different from what you will normally be asked to provide when you first visit our centre for medical checkup.
3. There is no major risk associated with your participation in this research. However, you may experience a minor bruise and/or temporary discomfort at the site of the blood draw and this risk is not more than you will be exposed to for having a blood draw at any health facility. You may also have to sacrifice part of your time in the morning at the study site taking part in the study.
4. How you will benefit from this study; we may find that you are suffering from any of the following conditions: asthma, bronchitis, emphysema and other lung function defect. In this case we will offer the necessary advice in your best interest. Your refusal to take part in this study will not affect our relationship with you in any way.
5. Results obtained from this study will strictly be confidential and if this study is published, you will not be identified in any way.
6. You may refuse to take part or withdraw from the study at any time without anyone objecting and without penalty or loss of any benefit to which you are otherwise entitled to.
7. Do you have any question now?
8. For any further enquiry during the period of your participation contact Mr. Isaac Ennin on telephone No. 0244694205 or come to the Medical School Dept of Physiology at Korle-Bu

**Consent**

I have fully explained to all the subjects the nature and purpose of the above study and all that are involve in its performance. I have answered and will answer all the questions to the best of my performance.

Full name ISAAC EKOW ENNIN Signature………………… Date…………

You have been fully informed of the above described research study with its possible benefits and risks. Your signature or thumbprint below indicates that you are wiling to participate in this research study. You do not give up any of your legal rights by signing this consent document.

Full name of subject ------------------------------ Signature or thumbprint------------

### QUESTIONNAIRE

ID No -------------------------------------------- Sex: ---------

Height: --------- Age: ---------

Weight: ----------------------- Position of patient----------

Room temperature & pressure -------------------

Date of test: ---------- Time of test----------

1. **Medical History**

Do you often wheeze? Y/N

Do you frequently sneeze? Y/N

Do you frequently cough? Y/N

Do you have running nose often? Y/N

Do you have block nose often? Y/N

Do you have itching nose often? Y/N

Do you often have itching throat? Y/N

Do you have breathlessness after work? Y/N

Do you often cough at work or at home? Y/N

Do you often have catarrh or running nose at work or at home? Y/N

Do you have chest pain due to your work? Y/N

Do you have shortness of breathlessness at rest? Y/N

Do you have breathlessness after work? Y/N

Do you have loss of voice? Y/N

Does any family member or relative have asthma? Y/N

Have you ever been diagnosed with asthma? Y/N

Do you have any thoracic cage abnormalities? Y/N

Do you have any vertebral column abnormalities? Y/N

Do you have any neuromuscular diseases? Y/N

Do you have any pulmonary tuberculosis? Y/N

Do you have bronchial asthma? Y/N

Have you ever had chest surgery? Y/N

1. **Occupational data**

Number of years as wood worker: ----------

Number of working hours per day: ----------

Number of working days per week: ----------

Nature of work…………………………………..

### Does the nature of your work release any substances into the air? Y/N

Name of substance…………………………………..

Does it smell? Y/N

Do you find yourself inhaling this substance? Y/N

Do you always use a nose mask? Y/N

If NO why?………………………………

Do you drink alcohol? Y/N

If yes for how long? ………………………………

Do you smoke? Y/N

If yes for how long? …………………………………..

Have you being exposed to any other industry other than wood industry? Y/N

If yes what industry?……………………………………………..

**3. Status of respiratory symptoms on weekends**

Do they disappear Y/N

Do they improve Y/N

No change Y/N

**4. Status of respiratory symptoms on vacations (more than one week)**

Do they disappear Y/N

Do they improve Y/N

No change Y/N

If they disappear or improve, after how many days? ……………………………

**5. Timing of respiratory symptoms in relation to work**

Interval between onset of work and onset of symptoms ………………

Persistence or reappearance of symptoms on return to home ……………………

Onset of symptoms only on returning home …………………………………….

Change of timing of symptoms over time………………………

**(Modified from the British Medical Research Council questionnaire on respiratory symptoms, 1960)**
